# Ancient Records and Modern Research on the Mechanisms of Chinese Herbal Medicines in the Treatment of Diabetes Mellitus

**DOI:** 10.1155/2015/747982

**Published:** 2015-03-01

**Authors:** Hai-ming Zhang, Feng-xia Liang, Rui Chen

**Affiliations:** ^1^Department of Traditional Chinese Medicine, Union Hospital, Tongji Medical College, Huazhong University of Science and Technology, No. 1277 Jiefang Street, Wuhan, Hubei 430022, China; ^2^Department of Acupuncture and Moxibustion, Hubei University of Traditional Chinese Medicine, No. 1 Tanhualin Street, Wuhan, Hubei 430060, China

## Abstract

Over the past decades, Chinese herbal medicines (CHM) have been extensively and intensively studied through from both clinical and experimental perspectives and CHM have been proved to be effective in the treatment of diabetes mellitus (DM). This study, by searching ancient records and modern research papers, reviewed CHM in terms of their clinical application and principal mechanism in the treatment of DM. We summarized the use of CHM mentioned in 54 famous ancient materia medica monographs and searched papers on the hypoglycemic effect of several representative CHM. Main mechanisms and limitations of CHM and further research direction for DM were discussed. On the basis of the study, we were led to conclude that TCM, as a main form of complementary and alternative medicine (CAM), was well recorded in ancient literatures and has less adverse effects as shown by modern studies. The mechanisms of CHM treatment of DM are complex, multilink, and multitarget, so we should find main hypoglycemic mechanism through doing research on CHM monomer active constituents. Many CHM monomer constituents possess noteworthy hypoglycemic effects. Therefore, developing a novel natural product for DM and its complications is of much significance. It is strongly significant to pay close attention to CHM for treatment of DM and its complications.

## 1. Introduction

Diabetes mellitus (DM), including type 1 and type 2, has become epidemic worldwide [1–3], and its incidence has been on rise year by year [4]. Previous reports have demonstrated that overweight, especially obesity at younger ages, substantially increases the risk for DM [1, 5–8]. The finding is consistent with the description in the “Medical Classic of the Yellow Emperor,” the earliest monumental work on the traditional Chinese medicine (TCM) dating back to the Warring States Period (about 446 B.C.−221 B.C.). DM increases the risk for micro- and macrovascular complications and premature death and poses tremendous socioeconomic burden [2, 4, 9]. In spite of the introduction of insulin and other hypoglycemic agents, so far, no treatment protocols can achieve a complete cure. Moreover, the side effects of these drugs, which are substantial and inevitable, present another challenge.

Complementary and alternative medicine (CAM) have been extensively used in modern times. TCM, as a main form of CAM, has been proved to be effective for the treatment of DM with relatively less side effects in China and beyond [10, 11]. Some hypoglycemic drugs of plant origin have been approved for clinical use by the regulatory authorities in China, such as* Yusanxiao*,* Yijin*, and* Kelening*, among others [12].

The mechanisms of Chinese herbal medicines (CHM) in the treatment of DM have been extensively and intensively studied from biological, immunological, and phytochemical perspectives and great advances have been made in the past decades. This paper reviewed records or descriptions concerning the use of CHM for treatment of DM in ancient Chinese literatures (before 1920 A.D.) and the modern papers on the mechanisms of CHM treating DM. We also compared the CHM used in ancient and modern times, examined the limitations of CHM for treating DM, and discussed the future research trend.

## 2. Ancient Records on Treatment of DM with TCM

Our search of literatures of TCM (before 1920 A.D. or earlier) failed to find the term “DM.” We found a plenty of records or descriptions about “*Xiao Ke*,” which, in terms of epidemiology, symptoms, etiology, pathogenesis, and treatment, mimicked those of DM. And it is generally accepted that “*Xiao Ke*” mentioned in ancient Chinese literature is similar to DM of modern medicine [13]. On basis of this assumption, in this paper, we used DM interchangeably with “*Xiao Ke*” for the convenience of discussion though they are not strictly equivalents in a number of ways.

### 2.1. Terminology, Epidemiology, Symptoms, Etiology, and Pathogenesis of “*Xiao Ke*”

#### 2.1.1. Name

In TCM, “*Xiao Ke*” refers to a cluster of clinical symptoms, including polydipsia, polyphagia, polyuria, emaciation, glucosuria, and fatigue. As aforementioned, “*Xiao Ke*” is a general term for a condition that resembles DM in terms of symptoms. DM classically was divided into three types: upper, middle, and lower “*Xiao Ke*.” The upper type (*Shang Xiao*) is characterized by excessive thirst, the middle type (*Zhong Xiao*) by excessive hunger, and the lower type (*Xia Xiao*) by excessive urination [13]. By searching “*Xiao Ke*,” we retrieved a large number of records concerning “*Xiao Ke*” in ancient TCM literatures.

#### 2.1.2. Epidemiology

The earliest mention of “*Xiao Ke*” was in the “Medical Classic of the Yellow Emperor.” The book described that the “*Xiao Ke*” was mostly found in wealthy, obese individuals who liked food rich in oil or fat and in influential officials who were on pills or “*Dan,*” as it was termed in the book, a mineral-based synthetic drug, which ancient people believe to be able to make them achieve longevity.

#### 2.1.3. Symptoms

The symptoms can be categorized into two groups: general symptoms and complications. The general symptoms include polydipsia, polyphagia, polyuria, glucosuria, emaciation, dry mouth, hunger, emptiness of the stomach, and frequent urination. And complications include diabetic foot, diabetic retinopathy, lung tuberculosis, diabetic impotence, and diabetic nephropathy. Obviously, those symptoms and complications are extremely similar to DM, as shown in Table 1.

#### 2.1.4. Etiology and Pathogenesis

According to the theory of TCM, the symptoms are essentially caused by “*Yin Xu*” (*Yin* deficiency) and “*Zao Re*” (dryness heat). In TCM there is a belief that* Yin* deficiency is the “*Ben”* (origin or root cause) and dryness heat is the “*Biao*” (symptoms or external manifestations). The* Ben* or root causes involve the invasion of exogenous pathogens, innate deficiency, intemperance in eating, abnormal emotional states (anger, anxiety, depression, distress, panic, and fear), excessive physical strains (mental or physical exertion and sexual intercourse), or propensity for abusing* Dan* medicines [11].* Yin* and* Yang* are two opposing aspects of things. For instance, cold, moist, night, structure, and downward mobility belong to* Yin* while heat, dryness, day, function, and upward mobility belong to* Yang* [14].

### 2.2. Treatment

We searched for the term “*Xiao Ke*” in more than 1,000 TCM ebooks included in Encyclopedia of TCM (Compact Disk, ISBN: 7-900377-49-2/R·8), published by Hunan Electronic and Audiovisual Publishing House. The database contained, among others, “*Bencao Gangmu *(Compendium of Materia Medica)”,* Puji fang, *and so forth.

#### 2.2.1. CHM

**Figure 1:**
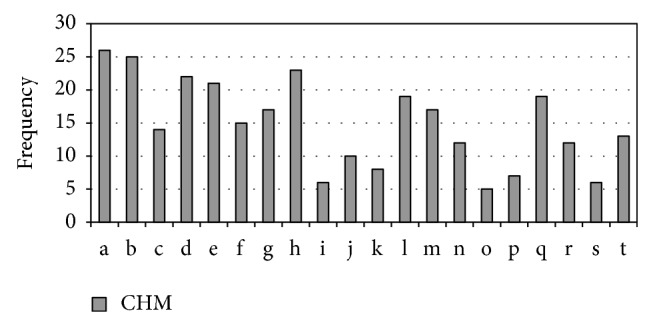
Frequency of heat-clearing (*Qing Re*) drugs for “*Xiao Ke*” mentioned in 54 monographs on Chinese materia medica. Heat-clearing drugs are of* Liang* (cold or cool) or bitter taste. a:* Pueraria lobata *(Willd.) Ohwi; b:* Trichosanthes kirilowii *Maxim.; c:* Fructus et semen trichosanthis kirilowii*; d:* Lemna minor *L.; e:* Gypsum fibrosum*; f:* Alisma orientale *(Sam.) Juz.; g:* Coptis chinensis *Franch.; h:* Anemarrhena asphodeloides *Bunge; i:* Lophatherum gracile *Brongn.; j:* Succus bambusae *(Recens); k:* Arctium lappa* L.; l:* Phragmites australis *(Cav.) Trin. ex Steud.; m:* Benincasa hispida *(Thunb.) Cogn.; n:* Phaseolus calcaratus *Roxb.; o:* Scutellaria baicalensis *Georgi; p:* Solanum lyratum *Thunb.; q:* Vitex negundo *var.* cannabifolia *(Siebold and Zucc.) Hand.-Mazz.; r:* Phellodendron chinense *C. K. Schneid.; s:* Gardenia jasminoides *J. Ellis; t:* Lycium chinense *Mill.

**Figure 2:**
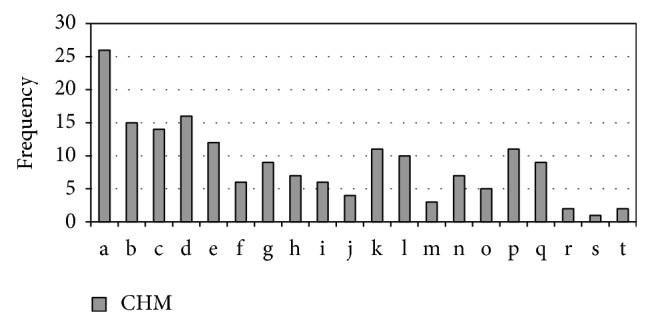
Frequency of* Yin*-nourishing (*Yang Yin*) and energy-replenishing (*Yi Qi*) drugs for “*Xiao Ke*” mentioned in 54 monographs on Chinese materia medica.* Yin*-nourishing and energy-replenishing drugsare of sweetish taste and are of cold (*Liang*) nature. a:* Lycium barbarum *L.; b:* Tussilago farfara *L.; c:* Poria cocos *(Schw.) Wolf; d:* Panax ginseng *C. A. Mey.; e:* Eleocharis dulcis *(Burm.f.) Trin. ex Hensch.; f:* Morus alba *L.; g:* Adenophora trachelioides *Maxim.; h:* Cannabis sativa *L.; i:* Ophiopogon japonicus *(Thunb.) Ker Gawl.; j:* Armeniaca mume *Siebold; k:* Asparagus cochinchinensis *(Lour.) Merr.; l:* Cuscuta chinensis *Lam.; m:* Achyranthes bidentata *Blume; n:* Coix lacryma-jobi *L.; o:* Astragalus membranaceus *(Fisch.) Bunge; p:* Polygonatum odoratum *(Mill.) Druce; q:* Rhus chinensis *Mill.; r:* Schisandra chinensis *(Turcz.) Baill.; s:* Lilium lancifolium *Thunb.; t:* Rehmannia glutinosa *Steud.

We also searched the database for Chinese crude drugs for treating “*Xiao Ke*.” The database contained only 54 monographs on Chinese materia medica. Most CHM treated “*Xiao Ke*” by “*Qing Re*” (clearing heat) (Figure 1), “*Yang Yin*” (nourishing* Yin*), and “*Yi Qi*” (replenishing vital energy) (Figure 2). The Latin names of CHM used in the paper were from the website http://www.theplantlist.org/ or http://www.wikipedia.org/.

#### 2.2.2. Foods

**Figure 3:**
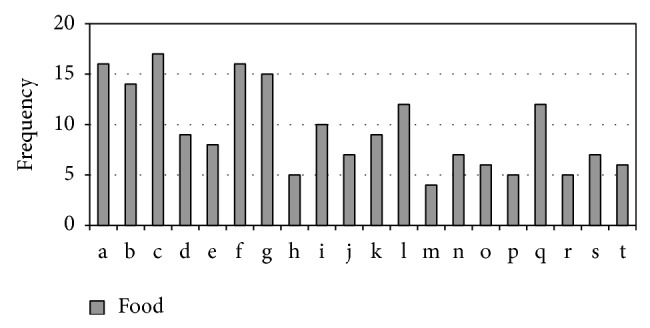
Frequency of meat, grains, fishes, and other food that help treat “*Xiao Ke*” mentioned in 54 monographs on Chinese materia medica. a: chicken; b: millet; c: barley; d: bamboo shoot; e: cony meat; f:* Benincasa hispida*; g: watershield leaf; h: mud eel; i: radish; j: foxtail millet seed; k: snail; l: cow's milk; m: goose meat; n: Charr; o: long surf clam; p: wheat; q: mung bean; r: Gallus black-bone silky fowl; s: hairy chestnut seed; t: giant gecko.

Besides, the monographs also mentioned some foods that help treat “*Xiao Ke*” in Figure 3.

## 3. Mechanisms by Which CHM Work on DM and Its Complications

We searched the databases of PubMed, Web of Science, MEDLINE, and CNKI and found that less research attention was paid to Chinese herbal compounds while most studies focused on a single herbal medicine.

The mechanisms of CHM in the treatment of DM have been extensively and intensively studied from biological, immunological, and phytochemical perspectives (Tables 2, 3, and 4).

## 4. Results

We found more than 40 CHM with hypoglycemic effect in ancient works and reviewed the mechanism of CHM lowering blood sugar. We were led to conclude that a number of CHM, including* Panax ginseng *C. A. Mey.,* Astragalus membranaceus *(Fisch.) Bunge, and* Lonicera japonica *Thunb., were used in ancient times and also nowadays. In addition, some CHM used for treating DM in ancient works have not been studied for hypoglycemic effect in modern times, such as* Lemna minor *L.,* Gardenia jasminoides *J. Ellis,* Eleocharis dulcis *(Burm.f.) Trin. ex Hensch., and* Achyranthes bidentata *Blume (Figures 1 and 2). These CHM may have potential to become drugs for the treatment of DM by further exploring their hypoglycemic effects. We also found that some foods were used for treatment of DM in ancient times, and their hypoglycemic effects have been confirmed nowadays [15, 16].

**Figure 4:**
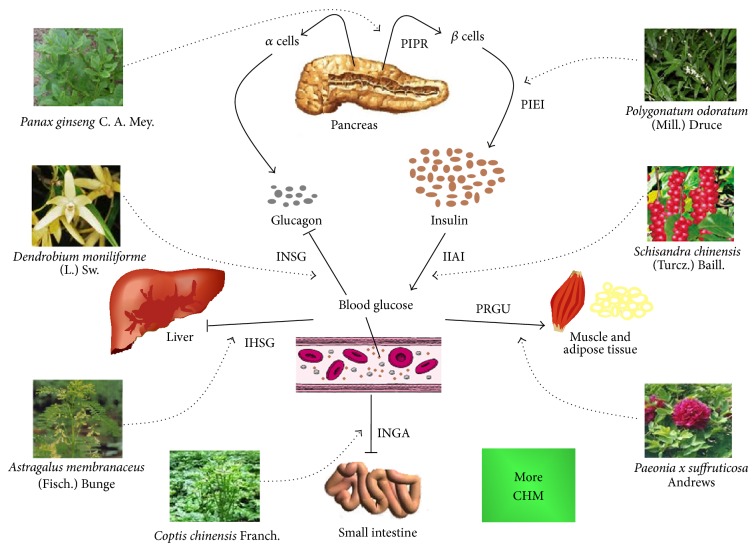
Main mechanisms of CHM working on DM. IIAI: CHM increase insulin sensitivity and ameliorate insulin resistance; PIEI: CHM promote insulin secretion and elevate serum insulin levels; INGA: CHM inhibit *α*-glucosidase activity; PIPR: CHM protect islet *β* cells and promote their regeneration; IHSG: CHM increase hepatic glycogen content and suppress gluconeogenesis; INSG: CHM inhibit the secretion of glucagon; PRGU: CHM promote the glucose uptake by adipose and muscular tissues. In the figure, seven CHM examples were given. CHM may involve a variety of hypoglycemic mechanisms, and only the main mechanism is mentioned in this figure. Dotted line means the possible ways in which CHM exert hypoglycemic effects. Solid lines show potential hypoglycemic mechanisms.

The mechanisms by which CHM treat diabetes include the following: (1) CHM increase insulin sensitivity and ameliorate insulin resistance; (2) CHM promote insulin secretion and elevate serum insulin levels; (3) CHM inhibit *α*-glucosidase activity; (4) CHM protect islet *β* cells and promote their regeneration; (5) CHM increase hepatic glycogen content and suppress gluconeogenesis; (6) CHM inhibit the secretion of glucagon; (7) CHM promote the glucose uptake by adipose and muscular tissues (Figure 4). Mechanisms of CHM treating diabetic complications include the following: (1) CHM control oxidative stress response, such as scavenging oxygen radicals, preventing lipid peroxidation, or inhibiting nitric oxide synthesis; (2) CHM regulate the activity of aldose reductase; (3) CHM block inflammatory response. Furthermore, CHM hypoglycemic effects are mainly based on IIAI, PIEI, INGA, PIPR, PRGU, and IHSG and fewer CHM are based on INSG.

## 5. Discussion

### 5.1. Limitations of Ancient Records and Modern Studies

First, some CHM can alleviate some symptoms of DM such as polydipsia, polyuria, and polyphagia. However, this does not necessarily mean that they are able to lower blood sugar. These drugs include* Phragmites australis *(Cav.) Trin. ex Steud.,* Alisma orientale *(Sam.) Juz., and Gypsum fibrosum. Second, toxicological studies on CHM were rarely conducted or no information was available on the toxicity of CHM. Fourth, many modern clinical and experimental studies on CHM were methodologically defective, which reduces their reliability and validity. Chen et al. and Li et al.'s results also stated this limitation [17, 18].

In addition, many modern clinical researches tended to focus on curative effects rather than underlying mechanisms. Although molecular biological, immunological, and phytochemical techniques have been widely applied to study the mechanism of CHM treating DM, the nature of many components or extracts was still not very clear.

### 5.2. Advantages of CHM in the Treatment of DM

Although CHM have many limitations, as aforementioned, the hypoglycemic effects of some CHM were well documented, and some can effectively ameliorate certain clinical symptoms of DM, such as polydipsia, polyuria, and polyphagia. A number of studies have shown that CHM or their extracts used in combination with western medicines work even better for the treatment of DM [19, 20]. For example,* Trigonella foenum-graecum *L. Saponin given together with sulphonylureas could effectively control the serum glucose, with few side effects, in DM patients whose serum glucose was not well controlled by oral administration of sulphonylureas [21].

### 5.3. Recommendations for Further Study of CHM for the Treatment of DM

CHM are increasingly used for the treatment of DM primarily because of increased awareness, on the part of patients and doctors, of their advantages, such as effectiveness, natural origin, and safety. However, in order to further extend their scope of application, the limitations of CHM should be avoided. More evidence-based clinical trials should be performed to substantiate the efficacy of CTM prescriptions and crude CHM for the treatment of DM. To confirm the effect of CHM on DM, larger-scale, multicentered, randomized, and controlled clinical trials are needed and statistical methods should be used in all clinical trials. Besides, the mechanisms of CHM and prescriptions should be examined at the molecular and cellular levels by fully taking advantage of the latest techniques, such as biochemical, biological, molecular biological, and immunological methods. Since adverse side effects associated with use of CHM, such as hepatotoxicity, nephrotoxicity and genotoxicity, were reported frequently, it is urgent to conduct toxicological studies on CHM. In order to achieve higher accuracy and better reproducibility, all studies on CHM should be conducted by following well-established and standardized procedures.

## 6. Conclusion

CHM used to and still play an important role in the treatment of DM in China and great progresses have been made over the last decades. A great many CHM monomer components possess antidiabetes actions. Therefore, it is of great significance to develop novel CHM for the treatment of DM and its complications. The underlying mechanism by which CHM treat DM are complicated and multifactorial and involve multiple organs; studying the effect of active monomer components of CHM might be a good starting point. It is strongly significant to pay close attention to CHM for treatment of DM and its complications.

## Figures and Tables

**Table 1:** A similar comparison of the symptoms of “*Xiao Ke*” and DM.

	Symptoms of “*Xiao Ke*” in Zhu Bing Yuan Hou Lun^a^	Symptoms of DM in Textbook of Internal Medicine [22]
General symptoms	Polydipsia; dry mouth and lips; polyphagia; hunger; emptiness of the stomach; frequent urination; polyuria; glucosuria; emaciation; adiposity; fatigue of limbs; mental fatigue; feverish dysphoria; itchy skin; hyperhidrosis; dizziness; sweet feeling in the mouth.	Polydipsia; thirst; polyphagia; hunger; polyuria; marasmus; obesity; sweet taste of urine; itchy skin; vulva pruritus; fatigue; lightheadedness.

Complications	Carbuncle and soreness; night blindness; internal oculopathy; lung tuberculosis; edema; precordial pain; pectoral stuffiness pain; apoplexy; coma; impotence; foot carbuncle-abscess; unsmooth defecation; diarrhea; anorexia; short breath; waist soreness; dizziness and tinnitus; pachylosis; whitish and turbid urine; muscle atrophy of the lower extremities; oliguria; nightly sweating; coolness of extremities.	Carbuncle and furuncle; diabetic retinopathy; pulmonary tuberculosis; diabetic cardiomyopathy; diabetic ketoacidosis; diabetic impotence; glaucoma; diabetic nephropathy; atherosclerosis; cerebral ischemic stroke; diabetic foot; constipation; diarrhea; myophagism; paralysis; oliguria; hyperhidrosis; hypohidrosis or anhidrosis; diabetic gastroparesis.

^a^The “Zhu Bing Yuan Hou Lun”: a book describing causes and manifestations of diseases by Yuanfang Chao, a famous TCM doctor born about AD 550 and died in 630 A.D. in the Sui Dynasty.

**Table 2:** Main mechanisms of CHM treating DM and its complications by nourishing *Yin* (*Yang Yin*) and benefiting vital energy (*Yi Qi*).

Latin name	Family	Extracts or monomers	*In vivo/* *in vitro *	Models	Effective doses/doses range	Mechanisms	Toxic effect	References
*Liriope spicata* Lour.	Liliaceae	Crude polysaccharide, water extract	*In vivo *	BABL/c mice	100, 200 mg/Kg	IIAI	NO	[23]

*Ophiopogon japonicus *(Thunb.) Ker Gawl.	Liliaceae	Polysaccharide	*In vivo *	KKAy mice, C57BL/6J mice	75, 300 mg/Kg	IIAI	ND	[24]
		Polysaccharide	*In vivo *	Ob/ob mice	300 mg/Kg	IIAI	ND	[25]

*Astragalus membranaceus *(Fisch.) Bunge	Leguminosae	Polysaccharide	*In vivo *	KKAy mice, C57BL/6J mice	700 mg/Kg	IIAI	ND	[26]
		Polysaccharide	*In vivo *	C57BL/6J mice	100, 400 mg/Kg	PIPR	ND	[27]
		Polysaccharide	*In vivo *	Sprague-Dawley (SD) rats	700 mg/Kg	IHSG	ND	[28]
			*In vitro *	C2C12 cells	0.05–0.2 mg/mL		YES, <200 *µ*g/mL	
		Astragaloside IV	*In vivo *	SD rats	1, 5 mg/Kg	BLIR	ND	[29]
		Calycosin	*In vitro *	Human umbilical vein endothelial cells	0.01 *µ*mol	BLIR	ND	[30]

*Panax ginseng *C. A. Mey.	Araliaceae	Malonyl ginsenosides	*In vivo *	Wistar rats	50, 100 mg/Kg	IIAI	ND	[31]
		Ginsenoside Rh2	*In vivo *	Wistar rats	1 mg/Kg	PIEI	ND	[32]
		Ginsenoside	*In vitro *	SD rats islet	0.1–1 mg/mL	PIEI	ND	[33]
		Aqueous extract	*In vivo *	Goto-Kakizaki rats, Wistar rats	200 mg/Kg	PIEI, PIPR, PRGU	ND	[34]
		Ginsenoside Re	*In vivo *	SD rats	20 mg/Kg	BLIR	ND	[35]

*Panax pseudoginseng *Wall.	Araliaceae	Panax notoginoside	*In vivo *	Wistar rats	100, 200 mg/Kg	COSR	ND	[36]

*Poria cocos *(Schw.) Wolf	Polyporaceae	Crude extract	*In vivo *	C57BL/KsJ-db/db mice, C57BL/6J mice	50 mg/Kg	IIAI	ND	[37]
		Dehydrotumulosic acid, dehydrotrametenolic acid, pachymic acid, triterpenes			1, 5, 10 mg/Kg			

*Dioscorea oppositifolia *L.	Dioscoreaceae	Decocted water	*In vivo *	Wistar rats	4 mg/Kg	IIAI	ND	[38]
		Polysaccharose	*In vivo *	Kun Ming mice	4.5 g/Kg	RAAR	ND	[39]

*Schisandra chinensis *(Turcz.) Baill.	Schisandraceae	Lignan	*In vivo *	SD rats	200 mg/Kg	IIAI, IHSG, PRGU	ND	[40]
			*In vitro *	3T3-L1 adipocytes, Min6 cells, human embryo kidney 293 cells,	0.5, 5 *µ*g/mL			

*Ophiocordyceps sinensis *(Berk.) G. H. Sung, J. M. Sung, Hywel-Jones, and Spatafora	Clavicipitaceae	Polysaccharide	*In vivo *	BALB/c mice, SD rats	200, 400 mg/Kg	PIEI	ND	[41]
		solid-state fermented mycelium	*In vivo *	KK/HIJ mice	300 mg/Kg	PIPR	ND	[42]

*Cornus Officinalis *Siebold and Zucc	Cornaceae	Methanol extract	*In vitro *	BRIN-BD11 cells, H4IIE cells	0–25 *µ*g/mL	PIEI, PIPR, IHSG	YES, cytotoxicity	[43]
		Proanthocyanidins	*In vivo *	Wistar rats	20 mg/Kg	INGA	ND	[44]
			*In vitro *	*α*-Glucosidase	1.2–2.1 *µ*g/mL			

*Polygonatum odoratum * (Mill.) Druce	Liliaceae	Total flavonoids	*In vivo *	Kun Ming mice, SD rats	50, 100, 200 mg/Kg	PIEI	ND	[45]
		Flavonoid, saponin	*In vivo *	SD rats	500 mg/Kg	COSR, INGA	NO	[46]

*Atractylodes macrocephala *Koidz.	Compositae	Atractylenolide, amino acid	*In vivo *	Kun Ming mice	1.8 g/Kg	RAAR	ND	[39]

*Codonopsis pilosula * (Franch.) Nannf.	Campanulaceae	Saccharides, amino acid	*In vivo *	Kun Ming mice	4.5 g/Kg	RAAR	ND	[39]

*Panax quinquefolius *L.	Araliaceae	Ginsenoside	*In vitro *	Rat pancreatic *β* cell derived cell line, INS-1	5, 125, 250 *µ*g/*µ*L	PIPR, PIEI	ND	[47]

*Rehmannia glutinosa Steud. *	Scrophulariaceae	Catalpol	*In vivo *	Wistar rats	0.1 mg/Kg	IHSG	ND	[48]
		Catalpol	*In vitro *	THP-1 cells	100, 300, 500 *µ*mol	COSR, BLIR	NO	[49]

*Dendrobium moniliforme * (L.) Sw.	Punicaceae	Water extract	*In vivo *	NIH mice, SD rats	125, 250, 500, 1000 mg/Kg	INSG, IHSG, PIEI	ND	[50]

*Dendrobium chrysotoxum *Lindl.	Punicaceae	Polysaccharide	*In vivo *	BALB/c mice,	200, 500 mg/Kg	COSR	ND	[51]
			*In vitro *	Mouse splenocytes, Jurkat cell, MCF-7 cells	0–200 *µ*g/mL			

*Ganoderma lucidum * (Leyss. ex Fr.) Karst	Polyporaceae	Polysaccharides	*In vivo *	Albino Swiss mice	50, 100, 200 mg/Kg	PIPR, COSR	NO	[52]
			*In vitro *	Wistar rat islets	25–100 *µ*g/mL			

IIAI: CHM increase insulin sensitivity and ameliorate insulin resistance; PIEI: CHM promote insulin secretion and elevate serum insulin levels; INGA: CHM inhibit *α*-glucosidase activity; PIPR: CHM protect islet *β* cells and promote their regeneration; IHSG: CHM increase hepatic glycogen content and suppress gluconeogenesis; INSG: CHM inhibit the secretion of glucagon; PRGU: CHM promote the glucose uptake by adipose and muscular tissues. COSR: CHM control oxidative stress response, such as scavenging oxygen radicals, preventing lipid peroxidation, or inhibiting nitric oxide synthesis; RAAR: CHM regulate the activity of aldose reductase; BLIR: CHM block inflammatory response. NO means not toxic. ND means no data available. YES means toxic.

**Table 3:** Main mechanisms of CHM treating DM and its complications by clearing heat (*Qing Re*).

Latin name	Family	Extracts or monomers	*In vivo/* *in vitro *	Models	Effective doses/doses range	Mechanisms	Toxic effect	References
*Paeonia x suffruticosa *Andrews	Paeoniaceae	Paeonol	*In vivo *	Newborn Wistar rats	200, 400 mg/Kg	PRGU, INGA	ND	[53]
			*In vitro *	Intestinal brush border membrane vesicles, rat hepatoma cell line H4IIE, human skin fibroblasts cell line Hs68, mouse adipocytes 3T3-L1	0.01–1 mg/mL,			
		Polysaccharide-2b	*In vivo *	Wistar rats	60 mg/Kg	IIAI	ND	[54]
		Paeonoside, apiopaeonoside, 6-methoxypaeoniflori-genone	*In vitro *	Human HepG2 cells, HUVECs	1–20 *µ*mol	IHSG	NO	[55]

*Morus alba *L.	Moraceae	1-Deoxynojirimycin, polysaccharide	*In vivo *	ICR mice	150 mg/Kg	IHSG, PIPR	ND	[56]

*Momordica charantia *L.	Cucurbitaceae	Saponin fraction, lipid fraction	*In vivo *	Db/db mice	150 mg/Kg	IIAI	ND	[57]
		Protein extract	*In vivo *	Wistar rats	5, 10 mg/Kg	PIEI, PRGU	ND	[58]
			*In vitro *	3T3-L1 adipocytes, C2C12 cells	0.01 *µ*g/mL			
		Saponins, momordicine II, kuguaglycoside	*In vitro *	MIN6 *β* cells	0.01–0.125 *µ*g/mL	PIEI	NO	[59]
		Ethanolic extract	*In vivo *	Albino Wistar rats	150, 300 mg/Kg	PIPR, IHSG, PRGU	ND	[60]
		Aqueous extract	*In vivo *	Albino Wistar rats	150 mg/Kg	COSR	ND	[61]

*Pueraria lobata *(Willd.) Ohwi	Leguminosae	Puerarin	*In vivo *	SD rats	100, 200 mg/Kg	IIAI	ND	[62]
		Daidzein	*In vivo *	Kun Ming mice	2.3 g/Kg	INGA, RAAR	ND	[39]
		Puerarin	*In vitro *	Wistar rats islets	100 *µ*mol	PIPR, COSR	ND	[63]

*Trigonella foenum-graecum *L.	Leguminosae	Hydroalcoholic extract	*In vivo *	C57BL/6J mice	2 g/Kg	IIAI	ND	[64]
		Trigonelline	*In vivo *	Wistar rats	40 mg/Kg	COSR	ND	[65]
		Fenugreek seeds powder	*In vivo *	Albino rats	Powder 5% in rat food	BLIR	ND	[66]

*Gardenia jasminoides *J. Ellis	Rubiaceae	Geniposide	*In vivo *	C57BL/6J mice	200, 400 mg/Kg	IHSG	ND	[67]

*Rheum palmatum* L.		Emodin	*In vivo *	B6. V- Lep^ob^/Lep^ob^ mice	25, 50 mg/Kg	PRGU	ND	[68]
			*In vitro *	3T3-L1 adipocytes	3 *µ*mol/L			

*Acorus calamus *L.	Araceae	Crude ethanol extract	*In vivo *	Homozygous C57BL/Ks db/db mice	100 mg/Kg	IIAI	ND	[69]
			*In vitro *	L6 rat skeletal muscle cells	12.5, 25 *µ*g/mL			
		Ethyl acetate fraction	*In vivo *	ICR mice	400, 800 mg/Kg	PIEI, INGA	ND	[70]
			*In vitro *	HIT-T15 cell line	0.41 *µ*g/mL			

*Eriobotrya japonica *(Thunb.) Lindl.	Rosaceae	Cinchonain-Ib	*In vivo *	Wister rats	108 mg/Kg	PIEI	ND	[71]
			*In vitro *	Rat insulinoma cell line, INS-1 cells	0.032 mg/mL			

*Anemarrhena asphodeloides* Bunge	Liliaceae	Timosaponin, anemaran	*In vivo *	Kun Ming mice	1.8 g/Kg	INGA	ND	[39]
		Total saponins	*In vivo *	SD rats	200 mg/Kg	BLIR	ND	[72]

*Lonicera japonica *Thunb.	Caprifoliaceae	Chlorogenic acid, ginnol	*In vivo *	Kun Ming mice	2.3 g/Kg	RAAR	ND	[39]

*Coptis chinensis *Franch.	Ranunculaceae	Berberine chloride form	*In vivo *	Wistar rats,	125, 500, 250 mg/Kg,	INGA	ND	[73]
				Beagle dogs	80 mg/Kg			
			*In vitro *	Caco-2 cells	2.5, 10, 40 mg/L			
		Berberine	*In vitro *	SD rats ventricular myocytes	0.1–100 *µ*mol/L	COSR	ND	[74]
		Berberine	*In vivo *	Wistar rats	100, 200 mg/Kg	PIPR, COSR	ND	[75]
		Berberine	*In vivo *	C57BLKS/J-Lepr^db^/Lepr^db^ mice,	5 mg/Kg	IIAI	ND	[76]
				Wistar rats	380 mg/Kg			
			*In vitro *	3T3-L1 cells, L6 cells	5 *µ*g/mL			

*Potentilla discolor *Bunge	Rosaceae	Flavonoids, triterpenoids	*In vivo *	Wistar rats	369, 501 mg/Kg	PIPR, COSR	ND	[77]

*Artemisia sphaerocephala *Krasch.	Compositae	Artemisia sphaerocephala Krasch. gum	*In vivo *	SD rats	0.3%, 0.9%, 2.7% gum	IIAI, IHSG	ND	[78]

*Sophora flavescens *Aiton	Leguminosae	Oxymatrine	*In vivo *	Wistar rats	60, 120 mg/Kg	COSR, BLIR	ND	[79]

*Punica granatum *L.	Punicaceae	Methanolic extract	*In vivo *	Zucker diabetic fatty rats, Zucker lean rats	100–500 mg/Kg	INGA	ND	[80]
			*In vitro *	*α*-glucosidase	0.5–32 *µ*g/mL			

*Arctium lappa *L.	Compositae	Arctigenin	*In vivo *	C57BL/6J mice, B6. V-Lep^ob^/Lep^ob^ mice	200, 25 mg/Kg	IHSG, PRGU	ND	[81]
			*In vitro *	L6 myotubes	0.1–3 *µ*g/mL			

IIAI: CHM increase insulin sensitivity and ameliorate insulin resistance; PIEI: CHM promote insulin secretion and elevate serum insulin levels; INGA: CHM inhibit *α*-glucosidase activity; PIPR: CHM protect islet *β* cells and promote their regeneration; IHSG: CHM increase hepatic glycogen content and suppress gluconeogenesis; INSG: CHM inhibit the secretion of glucagon; PRGU: CHM promote the glucose uptake by adipose and muscular tissues. COSR: CHM control oxidative stress response, such as scavenging oxygen radicals, preventing lipid peroxidation, or inhibiting nitric oxide synthesis; RAAR: CHM regulate the activity of aldose reductase; BLIR: CHM block inflammatory response. NO means not toxic. ND means no data available. YES means toxic.

**Table 4:** Main mechanisms of CHM treating DM and its complications by *Wen Yang* (tonifying *Yang*) or *Huo Xue Hua Yu* (activating blood circulation and easing congestion).

Latin name	Family	Extracts or monomers	*In vivo*/ *in vitro *	Models	Effective doses/doses range	Mechanisms	Toxic effect	References
*Amomum xanthioides *Wall. ex Baker	Zingiberaceae	Aqueous ethanolic extract	*In vitro *	3T3-L1 adipocytes	0.02–0.5 mg/mL	PRGU, IIAI	ND	[82]

*Angelica hirsutiflora *Tang S. Liu, C. Y. Chao, and T. I. Chuang	Umbelliferae	Methanolic extract	*In vivo *	ICR mice,	10, 30 mg/Kg	PIEI	ND	[83]
			*In vitro *	HIT-T15 cells, human pancreatic islets	50–150 *µ*g/mL			

*Ramulus cinnamomi *	Lauraceae	Cinnamaldehyde, benzyl benzoate	*In vivo *	Kun Ming mice	1.4 g/Kg	COSR	ND	[39]

*Cinnamomum cassia *(Nees and T. Nees) J. Presl	Lauraceae	Cinnamaldehyde, cinnamyl acetate, cassioside	*In vivo *	Kun Ming mice	700 mg/Kg	COSR	ND	[39]

*Eucommia ulmoides *Oliv.	Eucommiaceae	Lignans	*In vivo *	Kun Ming mice	1.4 g/Kg	COSR	ND	[39]
		Water extract	*In vivo *	C57BL/KsJ-db/db mice	1.87 g/Kg	IHSG	ND	[84]

*Daemonorops draco *(Willd.) Blume	Arecaceae	Ethanol extract	*In vivo *	ICR mice	1.2 g/Kg	PIPR, COSR	NO	[85]
			*In vitro *	RIN-m5F cells	10–100 *µ*g/mL		<200 *µ*g/mL	

*Zingiber officinale *Roscoe	Zingiberaceae	Phenolic gingerol	*In vitro *	L6 rat myoblast	5–40 *µ*g/mL	PRGU	NO	[86]

*Acanthopanax senticosus *(Rupr. and Maxim.) Harms	Araliaceae	Hot water extract	*In vivo *	Db/db mice	500 mg/Kg	INGA	ND	[87]
			*In vitro *	Caco-2 cells	0.03–4 mg/mL			
		Polysaccharide	*In vivo *	Wistar rats	200 mg/Kg	COSR	ND	[88]

*Ephedra sinica *Stapf	Ephedraceae	L-Ephedrine, alkaloid	*In vivo *	BALB/c mice	0.0125 mg/mL,	PIPR	ND	[89]

*Carica papaya *L.	Caricaceae	Aqueous extract	*In vivo *	Wistar rats	0.75, 1.5 g/100 mL,	PIPR, COSR, IHSG	ND	[90]

*Terminalia chebula *Retz.	Combretaceae	Chloroform extract	*In vivo *	SD rats	Short term study, 100, 200, 300 mg/Kg	PIEI	ND	[91]
					Long term study, 300 mg/Kg			

*Epimedium brevicornum*Maxim.	Berberidaceae	Icariin	*In vivo *	SD rats	80 mg/Kg	COSR	ND	[92]

*Salvia miltiorrhiza *Bunge	Lamiaceae	Hydrophilic extract	*In vitro *	HMEC-1 cells, human microvascular endothelial cells	10 *µ*g/mL	COSR	ND	[93]

IIAI: CHM increase insulin sensitivity and ameliorate insulin resistance; PIEI: CHM promote insulin secretion and elevate serum insulin levels; INGA: CHM inhibit *α*-glucosidase activity; PIPR: CHM protect islet *β* cells and promote their regeneration; IHSG: CHM increase hepatic glycogen content and suppress gluconeogenesis; INSG: CHM inhibit the secretion of glucagon; PRGU: CHM promote the glucose uptake by adipose and muscular tissues. COSR: CHM control oxidative stress response, such as scavenging oxygen radicals, preventing lipid peroxidation, or inhibiting nitric oxide synthesis; RAAR: CHM regulate the activity of aldose reductase; BLIR: CHM block inflammatory response. NO means not toxic. ND means no data available. YES means toxic.
